# Transmission of *Rickettsia raoultii* and *Rickettsia massiliae* DNA by *Dermacentor reticulatus* and *Rhipicephalus sanguineus* (*s*.*l*.) ticks during artificial feeding

**DOI:** 10.1186/s13071-018-3075-2

**Published:** 2018-09-03

**Authors:** Emanuela Olivieri, Michiel Wijnveld, Marise Bonga, Laura Berger, Maria T. Manfredi, Fabrizia Veronesi, Frans Jongejan

**Affiliations:** 1 Department of Veterinary Medicine, University of Perugia, 06126 Perugia, Italy; 2 Utrecht Centre for Tick-borne Diseases, FAO Reference Centre for Ticks and Tick-borne Diseases, Faculty of Veterinary Medicine, Utrecht University, Yalelaan 1, 3584 CL Utrecht, The Netherlands; 3 Institute for Hygiene and Applied Immunology, Center for Pathophysiology, Infectiology and Immunology, Medical University of Vienna, Kinderspitalgasse 15, 1090 Vienna, Austria; 4 Department of Veterinary Medicine, Università degli Studi di Milano, 20133 Milan, Italy; 5 Vectors and Vector-borne Diseases Research Programme, Department of Veterinary Tropical Diseases, Faculty of Veterinary Science, University of Pretoria, Private Bag X04, Onderstepoort, 0110 South Africa

**Keywords:** *Rickettsia raoultii*, *Rickettsia massiliae*, *Dermacentor reticulatus*, *Rhipicephalus sanguineus*, Transmission, *In vitro* feeding, Silicone membranes

## Abstract

**Background:**

Tick-borne rickettsial pathogens are emerging worldwide and pose an increased health risk to both humans and animals. A plethora of rickettsial species has been identified in ticks recovered from human and animal patients. However, the detection of rickettsial DNA in ticks does not necessarily mean that these ticks can act as vectors for these pathogens. Here, we used artificial feeding of ticks to confirm transmission of *Rickettsia massiliae* and *Rickettsia raoultii* by *Rhipicephalus sanguineus* (*sensu lato*) and *Dermacentor reticulatus* ticks, respectively. The speed of transmission was also determined.

**Methods:**

An artificial feeding system based on silicone membranes were used to feed adult *R. sanguineus* (*s*.*l*.) and *D. reticulatus* ticks. Blood samples from *in vitro* feeding units were analysed for the presence of rickettsial DNA using PCR and reverse line blot hybridisation.

**Results:**

The attachment rate of *R. sanguineus* (*s*.*l*.) ticks were 40.4% at 8 h post-application, increasing to 70.2% at 72 h. *Rickettsia massiliae* was detected in blood samples collected 8 h after the *R. sanguineus* (*s*.*l*.) ticks were placed into the *in vitro* feeding units. *D. reticulatus* ticks were pre-fed on sheep and subsequently transferred to the *in vitro* feeding system. The attachment rate was 29.1 % at 24 h post-application, increasing to 43.6 % at 96 h. *Rickettsia raoultii* was detected in blood collected 24 h after *D. reticulatus* was placed into the feeding units.

**Conclusions:**

*Rhipicephalus sanguineus* (*s*.*l*.) and *D. reticulatus* ticks are vectors of *R. massiliae* and *R. raoultii,* respectively. The transmission of *R. massiliae* as early as 8 h after tick attachment emphasises the importance of removing ticks as soon as possible to minimise transmission. This study highlights the relevance of *in vitro* feeding systems to provide insight into the vectorial capacity of ticks and the dynamics of tick-borne pathogen transmission.

## Background

Spotted fever group (SFG) rickettsiae are obligate intracellular Gram-negative bacteria belonging to the genus *Rickettsia* and are recognised agents of emerging infectious diseases in humans [1]. In Europe, *Rickettsia raoultii* was recently identified as an SFG *Rickettsia* causing tick-borne lymphadenopathy (TIBOLA), *Dermacentor*-borne necrosis erythema and lymphadenopathy (DEBONEL) and scalp eschar neck lymphadenopathy (SENLAT) [2–5]. Symptoms may include an inoculation eschar, cervical lymphadenopathy, high fever, malaise, and headaches [6]. *Rickettsia raoultii* is transmitted by, and isolated from, *Dermacentor marginatus*, *D. reticulatus, D. nuttalli* and *D. silvarum* ticks [3, 7–9].

The risk of human and animal exposure to pathogens transmitted by *D. reticulatus* is increasing in Europe, as this tick expands to new geographical areas [10–14]. In recent case reports from several clinical centres in China, *R. raoultii* was found infecting patients who presented with a febrile illness or a painful rash [9, 15]. In western Siberia, a recent study of 273 patients who were suffering from suspected tick-borne diseases was screened for rickettsial DNA, and ten of the patients tested positive; in further sequencing, three of the ten patients tested explicitly positive for *R. raoultii* [16]. These findings indicate that *R. raoultii* is emerging in a large part of the Eurasian continent.

*Rickettsia massiliae* is an SFG *Rickettsia* species prevalent worldwide, transmitted by and isolated from *Rhipicephalus sanguineus* (*s*.*l*.) [17–22]. *Rickettsia massiliae* is identified as an agent causing human disease [17, 23, 24] that can present as a febrile illness with rash [17, 23] or inoculation eschar and neck lymphadenopathy [24]. Also, there is an indication that *R. massiliae* may cause disease in dogs [25]; DNA of *R. massiliae* has been detected in blood from a dog with a splenic disease [26].

Moreover, in recent studies, *R. raoultii* DNA has been detected in questing *Ixodes ricinus* and *Dermacentor silvarum* ticks [9, 27, 28]. Further studies are required to determine whether these tick species can transmit *R. raoultii*.

To gain a better understanding of the transmission dynamics of SFG rickettsiae, we used an *in vitro* feeding system wherein adult *D. reticulatus* and *R. sanguineus* (*s*.*l*.) ticks were fed through silicone membranes over a blood reservoir. To monitor the transmission of *R. raoultii* and *R. massiliae*, blood samples were collected at regular intervals and analysed by PCR combined with the reverse line blot (RLB) hybridisation assay. The speed of transmission was also determined.

## Methods

### Ticks

All ticks used in this study originated from tick colonies maintained for several generations in the acaridarium established at the Utrecht Centre for Tick-Borne Diseases (UCTD). *Dermacentor reticulatus* ticks were kept at 90% relative humidity; developing stages were kept at 25 °C and non-developing stages were stored at 12 °C. The colony of *D. reticulatus* originated from the vegetation near Rozenburg in the south-western part of the Netherlands [29]. *Rhipicephalus sanguineus* (*s*.*l*.) ticks originated from dogs in Greece and were kept at 20 °C and 90% relative humidity.

To verify the presence of *R. raoultii* and *R. massiliae* in ticks derived from the respective tick colonies, PCR followed by sequencing (Microsynth, Balgach, Switzerland) of the *ompA*, *ompB*, *sca4*, *gltA* and 16S rRNA genes, together with the 23S–5S IGS region, was carried out as described previously [30]. All consensus sequences were submitted to the GenBank database and are available under accession numbers MG521356-MG521367. Moreover, DNA was extracted from all ticks used in the *in vitro* feeding experiments and screened for rickettsial DNA using PCR-RLB hybridisation.

### *In vitro* feeding of ticks

Ticks were fed using silicone membranes as described previously [31], with some modifications [32, 33] and additional adjustments. Specifically, 250 ml bovine blood was collected from cattle which belonged to the Farm Animal Department, Faculty of Veterinary Medicine at Utrecht University. All animals were screened using PCR-RLB to confirm that they were free from any tick-borne pathogen. The blood was collected directly into a bottle containing heparin (final concentration 20 IU/ml). Gentamicin and glucose were immediately added to a final concentration of 5 μg/ml and 2 g/l, respectively. The blood was stored at 4 °C until further use. When the blood was used for *in vitro* feeding of ticks, adenosine triphosphate (ATP) was added to reach a final concentration of 0.51 μg/ml. The blood was then preheated to 37 °C and distributed into 6-well tissue culture plates at 3.1 ml blood/well.

Silicone membranes were prepared using a layer of household plastic film fixed onto a glass sheet with adhesive tape. Lens cleaning paper (Tiffen, USA) was fixed on top of the film layer. A mixture containing 15 g silicone glue (RTV-1 Elastocil E4, Wacker, Germany), 4.5 g silicone oil (DC 200, Sigma-Aldrich, Taufkirchen, Germany), 2.9 g hexane (Sigma-Aldrich) and 0.15 g of white colour paste (RAL 9010, Wacker) was prepared and a thin layer was spread evenly over the lens cleaning paper. Membranes were left to polymerise for at least 24 h with 97% humidity in a closed environment filled with sheep hair to give the membranes a typical host odour. The thickness of the prepared membranes was checked with a microcallipers, and only those between 70 μm and 100 μm thickness were used. Membranes of correct thickness were attached with silicone glue to feeding units constructed from Plexiglas tubing according to previously published specifications [31]. At the start of each experiment, the protective layer of plastic film was removed, and finely cut pieces of sheep hair were placed on top of the membrane within the feeding unit to reinforce sheep odour. During the study, it proved challenging to feed *D. reticulatus* using the silicone membranes. To increase the feeding success rate, *D. reticulatus* were pre-fed on sheep for four days. On day four, the ticks were manually removed using forceps and directly placed in feeding units so that they could re-attach and continue feeding as soon as possible. Feeding difficulties were not observed with *R. sanguineus* (*s*.*l*.) ticks and therefore selected ticks were used directly, without pre-feeding.

Depending on availability and the activity of ticks, we used a combination of five male and five female ticks per feeding unit when possible. We selected only ticks that showed questing behaviour or movement after stimulation with CO_2_. For *D. reticulatus* we used six feeding units. In units 1–5 we placed five female and five male ticks. Feeding unit 6 contained five female ticks that were left over. Due to a lack of active *R. sanguineus* (*s*.*l*.) ticks, we were only able to use four feeding units. In unit 1 we placed five female and five male ticks. In units 2 and 3 we placed solely male ticks, ten and 13, respectively. The last feeding unit, unit 4, contained five female and nine male ticks. After applying the ticks, the feeding units were carefully lowered into wells of a 6-well tissue culture plate containing 3.1 ml pre-warmed bovine blood supplemented as described above. Plates containing *D. reticulatus* ticks were incubated at 37 °C with 90% relative humidity and 4% CO_2_. Plates containing *R. sanguineus* (*s*.*l*.) were placed in a water bath at 37 °C with a 100% relative humidity.

To limit disturbance of *D. reticulatus* during feeding, and thus prevent premature tick detachment, the blood was changed at 24-h intervals instead of 12-h intervals as previously described [32]. When the blood was changed, the part of the membrane in contact with the blood was rinsed with sterile PBS before placing the unit in a well containing fresh prewarmed blood. Tick attachment rates were checked, and blood samples were taken after resuspending the sedimented blood cells and collecting 1 ml of blood per well. The *D. reticulatus* ticks were allowed to feed for a total of 96 h.

For *R. sanguineus* (*s*.*l*.), the first blood change and sampling were at 8 h after placing the ticks in the feeding units. Other blood changes and samplings were at 24-h intervals, starting from the placement of the ticks in the feeding units. Final blood samples were taken at 72 h. Whereafter, the feeding was terminated.

All blood samples were stored at -20 °C. At the end of the artificial feeding experiments, ticks were stored in 70% ethanol until further use.

### Extraction of DNA from blood and *in vitro* fed ticks

Samples (200 μl) of collected blood were used for DNA extraction with the GeneJET Genomic DNA Purification Kit (Thermo Scientific, Karlsruhe, Germany), according to the manufacturer’s instructions. For DNA extraction from ticks, all were washed with demineralised water in an ultrasonic bath and then placed in individual tubes. Each tick was cut into smaller pieces using a sterile scalpel. Sectioned ticks were further disrupted in lysis buffer using the TissueLyser LT bead mill (Qiagen, Venlo, The Netherlands) with 7 mm metal beads at 50 oscillations for 3 min as described by the manufacturer. Subsequently, DNA was extracted using a GeneJET Genomic DNA Purification Kit. All the genomic DNA eluates were stored at -20 °C.

### PCR-reverse line blot hybridisation

To screen for rickettsial DNA, a PCR fragment (≈ 360bp) of the 16S rRNA gene was amplified with primers Rick-F1 (5'-GAA CGC TAT CGG TAT GCT TAA CAC A -3') and Rick-R2 (biotin 5'-CAT CAC TCA CTC GGT ATT GCT GGA-3') published by Christova et al. [34] and modified by Nijhof et al. [35]. Cycling conditions for PCR reactions in 25 μl volumes were as described by Giangaspero et al. [36]. The obtained amplicons were used in the RLB hybridisation assay to detect specific rickettsial species as previously described [34, 37]. Briefly, 10 μl PCR product was diluted with 150 μl 2× SSPE/0.1% SDS buffer to a final volume of 160 μl. The diluted PCR samples were denatured for 10 min at 100 °C and placed directly on ice. Samples were then centrifuged at 4 °C for 30 s and 11,000× *g*. Subsequently, 150 μl of each denatured PCR product was loaded into the slots of an MN45 miniblotter (Immunetics, Cambridge, MA, USA) and hybridised onto a membrane prepared with covalently bound specific oligonucleotide probes, for 60 min at 42 °C. Samples were removed by aspiration, and the membrane was removed from the miniblotter. To remove any falsely annealed PCR products, the membrane was washed twice with preheated 2× SSPE/0.5% SDS at 50 °C for 10 min with shaking. The membrane was then incubated at 42 °C with 50 ml 2× SSPE/0.5% SDS containing 5 μl streptavidin horseradish peroxidase conjugate at 500 U conjugate/ml (Streptavidin-POD conjugate, Roche, Woerden, The Netherlands) for 30 min under gentle shaking. To remove unbound conjugate, the membrane was washed twice with 2× SSPE/0.5% SDS at 42 °C for 10 min, followed by two washes with 2 × SSPE at room temperature for 5 min. Finally, membranes were incubated with ECL reagents (Amersham, Buckinghamshire, UK) and the chemiluminescence reactions were detected using an ECL hyperfilm (Amersham), which was developed using an automated X-ray developer.

## Results

### Sequencing of rickettsial DNA

PCR-RLB analysis of DNA extracted from a random selection of ticks from the tick colonies before the *in vitro* feeding assays confirmed the presence of *R. raoultii* in *D. reticulatus* ticks and *R. massiliae* in *R. sanguineus* (*s*.*l*.) ticks. The obtained rickettsial sequences were analysed using BLAST (http://blast.ncbi.nlm.nih.gov/). All rickettsial sequences originating from *D. reticulatus* ticks were similar (99–100%) to *R. raoultii* strain Khabarovsk genome (CP010969.1); all sequences obtained from *R. sanguineus* (*s*.*l*.) ticks were identical (100%) to *R. massiliae* strain AZT80 genome (CP003319.1) available in GenBank.

### Attachment of *D. reticulatus* and *R. sanguineus* (*s.l*.) during *in vitro* feeding

Following pre-feeding of *D. reticulatus* on sheep, the *in vitro* attachment rates were 29.1% after 24 h, 41.8% after 48 h and 43.6% after 96 h (Table 1).

**Table 1:** *In vitro* feeding of *Dermacentor reticulatus* ticks and transmission of *Rickettsia raoultii*

Time (h)^a^	Feeding unit	No. of ticks applied	Tick attachment		*R. raoultii* detected in blood^b^
			*n*	%	
24	1	10 (5m/5f)	3 (1m/2f)	30 (3/10)	-
	2	10 (5m/5f)	4 (2m/2f)	40 (4/10)	+
	3	10 (5m/5f)	2 (0m/2f)	20 (2/10)	-
	4	10 (5m/5f)	2 (2m/0f)	20 (2/10)	-
	5	10 (5m/5f)	3 (1m/2f)	30 (3/10)	-
	6	5 (0m/5f)	2 (0m/2f)	40 (2/5)	-
	Total	55	16	29.1 (16/55)	
48	1	10 (5m/5f)	5 (1m/4f)	50 (5/10)	-
	2	10 (5m/5f)	4 (2m/2f)	40 (4/10)	+
	3	10 (5m/5f)	4 (1m/3f)	40 (4/10)	-
	4	10 (5m/5f)	3 (2m/1f)	30 (3/10)	-
	5	10 (5m/5f)	3 (1m/2f)	30 (3/10)	+
	6	5 (0m/5f)	4 (0m/4f)	80 (4/5)	-
	Total	55	23	41.8 (23/55)	
72	1	10 (5m/5f)	5 (1m/4f)	50 (5/10)	+
	2	10 (5m/5f)	4 (1m/3f)	40 (4/10)	+
	3	10 (5m/5f)	4 (1m/3f)	40 (4/10)	-
	4	10 (5m/5f)	2 (1m/1f)	20 (2/10)	-
	5	10 (5m/5f)	4 (2m/2f)	40 (4/10)	-
	6	5 (0m/5f)	4 (0m/4f)	80 (4/5)	-
	Total	55	23	41.8 (23/55)	
96	1	10 (5m/5f)	5 (2m/3f)	50 (5/10)	-
	2	10 (5m/5f)	5 (1m/4f)	50 (5/10)	-
	3	10 (5m/5f)	4 (1m/3f)	40 (5/10)	-
	4	10 (5m/5f)	3 (2m/1f)	30 (5/10)	+
	5	10 (5m/5f)	3 (1m/2f)	30 (5/10)	+
	6	5 (0m/5f)	4 (0m/4f)	80 (4/5)	+
	Total	55	24	43.6 (24/55)	

^a^Sampling timepoints during *in vitro* feeding of *D. reticulatus*

^b^+: DNA of *Rickettsia raoultii* detected using PCR-RLB

*Abbreviations*: *m* male, *f* female

Attachment rates of *R. sanguineus* (*s*.*l*.) were 40.4% after 8 h, 70.2% after 24 h, 85.1% after 48 h and 70.2% after 72 h (Table 2).

**Table 2:** *In vitro* feeding of *Rhipicephalus sanguineus* (*s.l*.) ticks and transmission of *Rickettsia massiliae*

Time (h)	Feeding unit No.	No. of ticks applied	Tick attachment		*R. massiliae* detected in blood^a^
			*n*	%	
8	1	10 (5m/5f)	3 (1m/2f)	30 (3/10)	-
	2	10 (10m/0f)	8 (8m/0f)	80 (8/10)	-
	3	13 (13m/0f)	8 (8m/0f)	61.5 (8/13)	+
	4	14 (9m/5f)	0 (0m/0f)	0 (0/14)	-
	Total	47	19	40.4 (19/40)	
24	1	10 (5m/5f)	6 (2m/4f)	50 (5/10)	+
	2	10 (10m/0f)	9 (9m/0f)	40 (4/10)	-
	3	13 (13m/0f)	9 (9m/0f)	40 (4/10)	+
	4	14 (9m/5f)	9 (6m/3f)	30 (3/10)	+
	Total	47	33	70.2 (33/47)	
48	1	10 (5m/5f)	10 (5m/5f)	100 (10/10)	-
	2	10 (10m/0f)	9 (9m/0f)	90 (9/10)	-
	3	13 (13m/0f)	9 (9m/0f)	69.2 (9/13)	+
	4	14 (9m/5f)	12 (8m/4f)	85.7 (12/14)	+
	Total	47	40	85.1 (40/47)	
72	1	10 (5m/5f)	10 (5m/5f)	100 (10/10)	+
	2	10 (10m/0f)	8 (8m/0f)	80 (8/10)	-
	3	13 (13m/0f)	8 (8m/0f)	61.5 (8/13)	-
	4	14 (9m/5f)	7 (4m/3f)	50 (7/14)	+
	Total	47	33	70.2 (33/47)	

^a^+: DNA of *Rickettsia massiliae* detected using PCR-RLB

*Abbreviations*: *m* male, *f* female

### Detection of *R. raoultii* and *R. massiliae* DNA in blood samples

Pre-feeding of *D. reticulatus* on sheep prevented an accurate estimation of the earliest time frame wherein *R. raoultii* could be transmitted; we did, however, detect the transmission of this agent for the first time in an artificial feeding system. The blood sample taken 24 h after the ticks were applied to feeding unit #2 tested positive for *R. raoultii* DNA (Table 1), samples remained positive for up to 72 h post-tick application. Blood samples from unit #5 tested positive at 48 h and 96 h; samples from unit #1 were positive at 72 h, unit #4 at 96 h and unit #6 at 96 h (Table 1).

*Rhipicephalus sanguineus* (*s*.*l*.) ticks were more willing to feed through the silicone membranes and were less sensitive during the handling of the feeding units. This allowed us to change the blood meal and collect samples 8 h after the ticks were applied to the units. Thus, the earliest time point at which *R. massiliae* DNA was detected was 8 h after the ticks were placed in the feeding units (unit #3, Table 2). Samples from feeding unit #3 remained positive until 48 h. Blood samples from unit #1 tested positive at 24 h and 72 h; samples from unit #4 tested positive at 24 h, 48 h and 72 h (Table 2).

### PCR-RLB analysis of tick DNA after *in vitro* feeding

PCR-RLB analysis of extracted tick DNA revealed that 90.9% (50/55) of the *in vitro* fed *D. reticulatus* tested positive for *R. raoultii* and 40.4% (19/47) of *R. sanguineus* (*s*.*l*.) for *R. massiliae*.

## Discussion

Our experiments confirm the effectiveness of *in vitro* feeding assays using silicone membranes, although we were more successful with *R. sanguineus* (*s.l*.) than with *D. reticulatus* ticks. *In vitro* feeding systems using silicone membranes were initially developed for *Ixodes ricinus*, whereby attachment rates of 90% have been achieved [38–40]. Other tick species have also been adapted to feed *in vitro*, in particular, *Amblyomma americanum*, with attachment rates between 50% and 75% [41]; *Hyalomma dromedarii* and *H. anatolicum* with rates of 55% and 75%, respectively [42], and *Ixodes scapularis* ticks which reached an attachment rate of 45% *in vitro* [33].

The relatively low attachment rate of *D. reticulatus* ticks (29.1% after 24 h, up to 43.6% after 96 h) was also reported in a recent study conducted by Krull et al., wherein the average attachment rate was 31.6% [43]. In the same study, it was demonstrated that an increased CO_2_ level could act as a feeding stimulant and therefore be improving the attachment rate [43]. Further studies are required to enhance the *in vitro* feeding behaviour of *D. reticulatus* ticks.

The attachment rates of *R. sanguineus* (*s*.*l*.) were significantly higher than those of *D. reticulatus* ticks; namely, 70.2% after 24 h, up to 85.1% after 48 h. This was also observed in a study conducted by Fourie et al., wherein the attachment rate was as high as 72.5% after only 24 hours [32].

The observed differences in attachment and feeding behaviour of different tick species indicate that there is still much to learn about the behaviour biology of ticks, whereby feeding stimulants that work for one tick species do not necessarily work for another species. Further optimisation of feeding conditions is required as *in vitro* assays prove vital for studying the vectorial capacity of ticks without the influence of laboratory animals.

Although the vectorial capacity of *D. reticulatus* and *R. sanguineus* (*s*.*l*.) regarding the respective *Rickettsia* species has already been documented [18, 29, 30, 44–46], here we demonstrate for the first time that both rickettsial pathogens are transmitted by *in vitro* feeding vector ticks. The detection of *R. raoultii* and *R. massiliae* DNA indicated that both organisms were transmitted *in vitro*, although in theory, it could have been DNA only. *Rickettsia massiliae* was detected as early as 8 h after the ticks were placed within the feeding units, confirming that transmission of tick-borne microorganisms can occur at a relatively early stage after tick attachment. We were unable to determine whether *R. raoultii* could also be transmitted at an early stage after tick attachment, because of the necessity to pre-feed *D. reticulatus* ticks on sheep. We did, however, observe *R. raoultii* transmission 24 h after the ticks were placed in the feeding units, proving the efficacy of the artificial feeding system in studying the transmission dynamics of tick-borne pathogens. Throughout the artificial feeding assays, we observed ticks attaching and feeding, but rickettsial DNA was not always found in the blood of the respective wells. This might indicate that the ticks feeding at that specific time point were negative or had just started feeding, as we were unable to observe tick attachment and feeding behaviour continuously. Furthermore, ticks may have been disturbed while feeding on the silicone membrane and re-attached at a later time, or *Rickettsia*-positive ticks might have fed during different time periods in the same feeding unit. This could explain why feeding unit #1, in the *R. sanguineus* (*s*.*l*.) feeding assay, was positive for *R. massiliae* DNA at 24 h, negative at 48 h and became positive again 72 h after tick application (Table 2).

Finally, our results further confirm the importance of removing ticks as soon as possible to minimise the risk of infection with tick-borne bacteria. More recently, *Ehrlichia canis* was transmitted as soon as 6 hours after tick attachment *in vivo* on dogs and within 8 hours by *in vitro* feeding *R. sanguineus* (*s*.*l*.) ticks [32]. The relatively short period observed for rickettsiae to be transmitted might be because of the presence of the bacteria within the salivary glands [47] and haemolymph [30, 48] before feeding. This contrasts with other bacteria such as *Borrelia burgdorferi* (*s*.*l*.) which are present in the midgut before feeding. The earliest transmission period for *Borreliella burgdorferi* (*s*.*l*.) is witnessed as early as 17 hours after attachment of infected *I. ricinus* ticks [49]. This is due to that the spirochetes are attached to the midgut of ticks and require external stimuli to pass through the midgut into the haemolymph and reach the salivary glands before transmission can occur [50].

## Conclusions

In our study, *D. reticulatus* and *R. sanguineus* (*s*.*l*.) ticks successfully fed in artificial feeding systems. *Rickettsia massiliae* DNA was detected as early as 8 h after the ticks were applied to the feeding units; *Rickettsia raoultii* DNA was detected 24 h after (pre-fed) ticks were placed within the feeding units. The early transmission time of tick-borne rickettsial species emphasises the importance of removing ticks as soon as possible and the use of tick repellents to minimise the risk of exposure to tick-borne microorganisms.

## Abbreviations

ATP: adenosine triphosphate
DEBONEL: *Dermacentor*-borne necrosis erythema lymphadenopathy
RLB: reverse line blot
SDS: sodium dodecyl sulphate
SENLAT: scalp eschar neck lymphadenopathy
SFG: spotted fever group
TIBOLA: tick-borne lymphadenopathy
